# What Is the Molecular Signature of Mind–Body Interventions? A Systematic Review of Gene Expression Changes Induced by Meditation and Related Practices

**DOI:** 10.3389/fimmu.2017.00670

**Published:** 2017-06-16

**Authors:** Ivana Buric, Miguel Farias, Jonathan Jong, Christopher Mee, Inti A. Brazil

**Affiliations:** ^1^Brain, Belief, and Behaviour Lab, Centre for Psychology, Behaviour, and Achievement, Faculty of Health and Life Sciences, Coventry University, Coventry, United Kingdom; ^2^Donders Institute for Brain, Cognition and Behaviour, Radboud University, Nijmegen, Netherlands; ^3^Centre for Applied Biological and Exercise Sciences, Faculty of Health and Life Sciences, Coventry University, Coventry, United Kingdom; ^4^Forensic Psychiatric Centre Pompestichting, Nijmegen, Netherlands; ^5^Collaborative Antwerp Psychiatric Research Institute, Antwerp University, Antwerp, Belgium

**Keywords:** gene expression, meditation, yoga, mind–body, nuclear factor kappa B, inflammation, stress, conserved transcriptional response to adversity

## Abstract

There is considerable evidence for the effectiveness of mind–body interventions (MBIs) in improving mental and physical health, but the molecular mechanisms of these benefits remain poorly understood. One hypothesis is that MBIs reverse expression of genes involved in inflammatory reactions that are induced by stress. This systematic review was conducted to examine changes in gene expression that occur after MBIs and to explore how these molecular changes are related to health. We searched PubMed throughout September 2016 to look for studies that have used gene expression analysis in MBIs (i.e., mindfulness, yoga, Tai Chi, Qigong, relaxation response, and breath regulation). Due to the limited quantity of studies, we included both clinical and non-clinical samples with any type of research design. Eighteen relevant studies were retrieved and analyzed. Overall, the studies indicate that these practices are associated with a downregulation of nuclear factor kappa B pathway; this is the opposite of the effects of chronic stress on gene expression and suggests that MBI practices may lead to a reduced risk of inflammation-related diseases. However, it is unclear how the effects of MBIs compare to other healthy interventions such as exercise or nutrition due to the small number of available studies. More research is required to be able to understand the effects of MBIs at the molecular level.

## Introduction

In the past two decades, mind–body interventions (MBIs; refer to Table A1 for a list of abbreviations frequently used throughout this paper) have been gaining empirical support and recognition by mental health professionals. While some MBIs, such as yoga, Tai Chi, and Qigong, have a strong physical component, others like meditation and mindfulness, breath regulation techniques, and the relaxation response (RR) are mainly sedentary. Despite the variability in these techniques, they all seem to produce various psychological benefits on healthy and clinical populations, such as the reduction of perceived stress [e.g., Ref. (1)], the alleviation of depression [e.g., Ref. (2)], decreases in anxiety [e.g., Ref. (3)], or to help in coping with a chronic medical disease [e.g., Ref. (4)]. However, it is less clear what are the mechanisms underpinning the self-reported benefits of MBIs. Neuroimaging studies suggest that MBIs increase gray matter in the brain regions related to emotion regulation, learning, memory, self-referential processes, and perspective taking (5–7). However, a recent meta-analysis on structural and morphological brain changes associated with one type of MBI (meditation) casts some doubt on the generalization of such results, as different techniques and length of practice are associated with different neural patterns (8).

The search for potential mechanisms of MBIs should not stop at the neural level. The development of gene expression analysis techniques in recent year makes this one important tool for psychologists to gain a deeper understanding of biological mechanisms that underpin, or interact with, psychological variables. Over the past decade, studies that implement gene expression analysis in MBIs research have begun to appear. In addition to being an objective measure of evaluating and comparing the effectiveness of MBIs, the analysis of gene expression changes has considerably theoretical value, as it reveals the underlying mechanisms of the psychological and physical effects of MBIs.

In this systematic review, we will explore (1) if MBIs can affect physical health by causing observable molecular changes in the form of differential gene expression and (2) what are the molecular changes underpinning psychological benefits in MBIs. By implementing a biological approach to the study of MBIs, there is an opportunity to fill a crucial gap concerning the underlying mechanisms that give rise to their reported beneficial effects. We have included a range of MBIs in our analysis, such as mindfulness and other forms of meditation, yoga, RR, Tai Chi, and Qigong, all interventions for which there is evidence suggesting similar beneficial effects on mental and physical health (9).

Below we start by outlining the principles of gene expression analyses and how they have been applied to MBIs; then we move into a systematic review of the evidence for their effects on gene expression, and what changes in gene expression underpin the psychological benefits of MBIs. Finally, we will discuss the implications of the reviewed studies, their limitations, and offer guidance for future studies.

### Gene Expression and Bioinformatics Analysis in MBIs

Gene expression detection techniques produce an enormous amount of quantitative data—a long list of genes. But because genes are most often team players—they work together as a network to produce an observable trait or a measurable biological function—a long list of genes is hard to contextualize and interpret. To make matters more complicated, some genes regulate the activity of other genes. One way to deal with this is to start with statistical analysis, followed by bioinformatics analyses; this one is used to identify which of the genes are in the same pathway (i.e., network) and, therefore, have the same function.

The most common bioinformatics analysis in MBIs research is the Transcription Element Listening System (TELiS). This analysis will assess which transcription factor is regulating gene expression amid a set of genes. It does so by scanning the promoters for transcription factor binding motifs that are overrepresented, in relation to their usual prevalence across the genome (10). In the context of research on MBIs, the most researched transcription factors are those that have been associated with stress and inflammation. The key transcription factor is the nuclear factor kappa B (NF-κB), which is produced when stress activates the sympathetic nervous system (SNS) (11). NF-κB translates stress into inflammation by changing the expression of genes which code for inflammatory cytokines (12). Lower activity of NF-κB suggests reduced inflammation.

### Understanding Stress and Conserved Transcriptional Response to Adversity (CTRA)

Stress can be regarded as a bodily response to events that are perceived as a threat or a challenge. This response may precipitate a health risk when stress is severe or it occurs over a long period of time without adequate coping mechanisms. It has been found that exposure to severe stressors can have a profound influence on the body and can lead to detrimental changes in its biology, such as reduced gray matter in several brain regions (13). The effects of stress go beyond the brain and can be found in our genes in a form of a CTRA (14). CTRA is a common molecular pattern that has been found in people exposed to different types of adversities, such bereavement (15), cancer diagnosis (16), trauma (17), and low socioeconomic status (18). The primary characteristic of CTRA is the upregulation of pro-inflammatory genes leading to major inflammation at the cellular level (19). While acute inflammation is a short-lived adaptive response of our body, which increases the activity of the immune system to fight injury or infections, chronic inflammation is maladaptive because it persists when there is no actual threat to the body. Chronic inflammation is associated with increased risk for some types of cancer, neurodegenerative diseases, asthma, arthritis, cardiovascular diseases, and psychiatric disorders (e.g., depression and posttraumatic stress disorder) (20, 21). The secondary characteristic of CTRA is the downregulation of antiviral and antibody-related genes, which is associated with susceptibility to viral infections, such as herpes simplex viruses (22), HIV-1 (23), Epstein–Barr virus (24), cytomegalovirus (25), and the Kaposi’s sarcoma (26). Given this, the CTRA is considered to be a molecular signature of chronic stress.

To understand the impact of environmental factors on the body’s immune system through CTRA, we first need to unpack the underlying molecular mechanisms. Consider the path linking a stressful event to an observable psychobiological change, such as the onset of depression. Slavich and Irwin (19) suggested that the environmental stressor, which might be a physical or social threat, will first activate brain regions associated with pain; then, it will project into lower regions that modulate inflammation *via* the hypothalamus–pituitary–adrenal (HPA) axis and the SNS.

In the first stage of modulation, the SNS initiates the production of the neuromodulators epinephrine and norepinephrine. These will, in turn, promote inflammation by activating the production of molecules called transcription factors that then bind to and activate pro-inflammatory genes and translate them into proteins cytokines that can inhibit or initiate inflammation. Cytokines will travel back to the brain and initiate symptoms of depression [e.g., low mood, fatigue, and anhedonia (27)].

In the second stage of modulation, the HPA initiates the production of metabolic agents (glucocorticoids) and the neurotransmitter acetylcholine that, in normal conditions, suppress inflammation. In the case of long-term stress, the body adapts to their continuous secretion and becomes less sensitive to their anti-inflammatory effects. These processes lead to CTRA and, if this condition is maintained for years, there is a high risk of inflammation-related diseases, infection, accelerated biological aging, and early mortality. It is likely that CTRA played an important role in our hunter-gatherer prehistory, as it linked fight-or-flight response with pro-inflammatory gene expression that provided protection when there was a higher risk of bacterial infections from wounds (19). This immune response might have been adaptive back then, but, in today’s modern societies where stress is primarily the result of psychological threats, this response is maladaptive as it promotes inflammation-related diseases, both psychiatric and medical (28).

## Methods

### Criteria for Considering Studies for this Review

**Figure 1:**
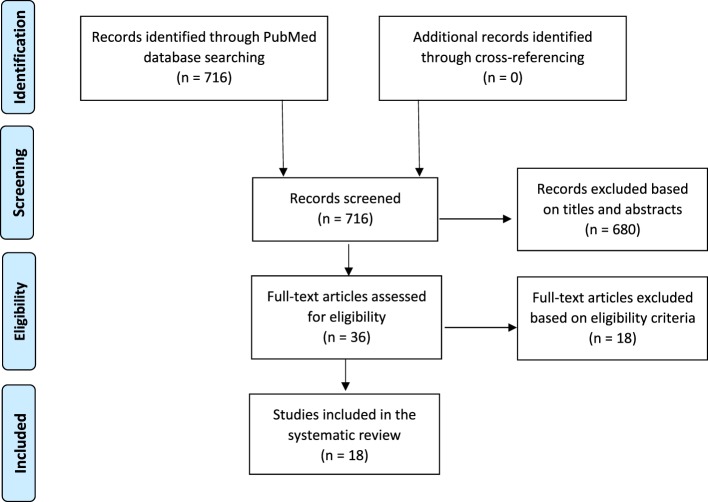
PRISMA flow chart: the process of selecting relevant studies.

We will now review studies on MBIs (mindfulness, yoga, RR, Tai Chi, and Qigong) that include gene expression analysis as an outcome measure, in order to assess the evidence for their effects on gene expression, and what changes in gene expression underpin the psychological benefits of MBIs. Studies were identified by searching PubMed through September 2016 using the following combination of keywords: *(meditation OR mindfulness OR relaxation response OR yoga OR tai chi OR Qigong) and (gene expression OR microarray OR transcriptome)*. A total of 716 articles were returned and their titles and abstracts were screened (see Figure 1). We excluded studies that did not meet the following eligibility criteria:

1. The population studied should only contain adults.
2. Both clinical and non-clinical samples were allowed (for example, students, cancer patients, and caregivers) and studies with all sample sizes were included.
3. Studies with experienced practitioners or non-experienced practitioners were allowed, making both cross-sectional and longitudinal studies eligible.
4. Gene expression changes had to be one of the outcome variables (any number of analyzed genes, cell type and any gene expression technology were eligible).
5. The independent variables had to be any type of MBI.
6. Articles should be written in English.
7. Only research papers were included. Review papers, meta-analyses, commentaries, and conference proceedings were excluded.

The screening narrowed the search results to 18 articles (Figure 1). The references of included articles were searched to identify other relevant articles, but no additional studies were found. Therefore, a total of 18 studies with 846 participants were included in this review (Table 1). Three studies had cross-sectional designs: they compared experienced practitioners to non-practitioners. Eight studies were longitudinal: they monitored changes over time that happened as one learns an MBI. Two studies measured immediate effects of a meditation session in experts, and the three remaining studies have elements of both cross-sectional and longitudinal designs. In the next section, we will describe the rationale for each of the included studies, along with the procedures employed and their results. We divided the studies across three sections, based on the research design, and taking into account their chronological order to highlight how complexity increases as gene expression technologies advance.

**Table 1:** A summary of the results of MBI and gene expression.

Study	Research design	Type of population (experimental group sample size)	Control group type (size)	Meditation or meditation-related type of practice	Practice frequency, training time	Gene expression technology (bioinformatics analysis)	Cell type	Biological outcome	Psychological and other outcomes
Li et al. (29)	CS	Experienced practitioners (*n* = 6)	Naïve (*n* = 6)	Qigong	60–120 min/day, 1–5 years	Genome-wide: Affymetrix Human Genome U95 (N/A)	Peripheral blood neutrophils	Genes related to: apoptosis− Cell metabolism− Immune regulation+	N/A
Sharma et al. (30)	CS	Experienced practitioners (*n* = 42)	Naïve (*n* = 42)	Sudarshan Kriya (breath regulation)	>60 min/day, at least 1 year	RT-PCR of 9 genes (N/A)	Peripheral blood lymphocytes	Genes related to: oxidative stress ns, DNA damage ns, cell cycle control ns, aging ns, apoptosis+	N/A
Kumar and Balkrishna (31)	CS	Leukemia patients (*n* = 8)	Naïve (*n* = N/A)	Pranayama (breath regulation)	N/A	Genome-wide: Expression Array System of Applied Biosystems (N/A)	Peripheral blood lymphocytes	Genes related to: immune regulation+ Apoptosis+	N/A
Creswell et al. (32)	LG	Normal older adults (*n* = 20)	Wait list (*n* = 20)	Mindfulness-based stress reduction, 8 weeks	30 min/day, 8 weeks	Genome-wide: Illumina HT 12 BeadChip (TELiS, NF-κB)	PBMC	Transcription factors: NF-κB−; Proteins: CRP− IL-6 ns	Loneliness−
Black et al. (33)	LG	Dementia caregivers (*n* = 20)	Relaxing music (*n* = 20)	Kirtan Kriya Meditation	12 min/day, 8 weeks	Genome-wide: Illumina HT 12 BeadChip (TELiS, NF-κB, and IRF)	PBMC	NF-κB−, IRF+ IL-6 ns, IL-8 ns, IL1B ns, TNF ns	Depression−, mental health+
Irwin et al. (34)	LG	Breast cancer survivors with insomnia (*n* = 40)	Cognitive behavioral therapy (*n* = 40)	Tai Chi	2 h/week, 12 weeks	Genome-wide: Illumina HT 12 BeadChip (TELiS, NF-κB)	PBMC	NF-κB− CRP ns IL-6 ns TNF−	N/A
Bower et al. (35)	LG	Breast cancer survivors with fatigue (*n* = 16)	Health education (*n* = 15)	Iyengar yoga	90 min/twice a week, 12 weeks	Genome-wide: Illumina HT 12 BeadChip (TELiS: NF-κB, GR, and CREB)	PBMC	NF-κB−, CREB−, GR+ Cortisol ns, sTNFRII− IL-1RA ns CRP ns IL-6 ns	N/A
Bower et al. (36)	LG	Breast cancer survivors (*n* = 39)	Wait list (*n* = 32)	Mindful awareness practice	2 h/week, 6 weeks	Genome wide: Illumina HT 12 BeadChip(TELiS: NF-κB, GR, CREB, IRF)	PBMC	NF-κB− GR + IRF + CREB ns sTNFRII ns CRP ns IL-6 ns	Stress−. Fatigue−, sleep disturbance−, hot flashes−, depression− (marginal), positive affect+, peace and meaning+, intrusive thought ns, fear of recurrence ns
Duraimani et al. (37)	LG	Hypertensive adults (*n* = 24)	Extensive health education (*n* = 24)	Transcendental meditation	40 min/day, 16 weeks	2 genes related to telomeres: RT-PCR	Whole blood	hTR ns, hTERT ns, Telomere length ns	Blood pressure ns, healthy lifestyle ns, anger ns
Irwin et al. (34)	LG	Older adults with insomnia (*n* = 49)	Cognitive behavioral therapy for insomnia (*n* = 49), sleep education (*n* = 25)	Tai Chi	2 h/week, 16 weeks	Genome-wide: Illumina HT 12 BeadChip (TELiS: NF-κB, CREB, GR, AP, IRF1, IRF2)	PBMC	NF-κB−, CREB−, GR+, AP−, IRF1 ns, IRF2 ns; CRP ns TLR4−	N/A
Kuo et al. (38)	LG	Irritable bowel syndrome and inflammatory bowel disease (*n* = 48)	None	Relaxation response–mind–body intervention	20 min/day, 9 weeks	Genome-wide: Affymetrix HG U133 Plus (Interactive network analysis: NF-κB)	PBMC	NF-κB− ESR ns, CRP ns	Quality of life+, symptoms of IBS/IBD−, pain catastrophizing−, pain interference with daily life ns, anxiety−
Ho et al. (39)	LG	Caregivers (*n* = 25)	None	MBSR	1.5 h/week (8 weeks)	Genome-wide: Affymetrix HuGene 1.0 ST arrays (WebGestalt2)	PBMC	Genes related to inflammation−, Stress response−, Depression−	Psychological stress+, mindfulness+
Ravnik-Glavač et al. (40)	RR	Experienced practitioners (*n* = 2)	None	Buddhist forms of meditation	N/A, more than 23 years of experience	Genome-wide: Affymetrix HG U133 Plus, ArrayStar Human LncRNA Array (Gene Enrichment Analysis)	PBMC	Genes related to metabolism and cell cycle processes−, immune system ns, apoptosis ns, stress response ns EEG: theta+, alpha+	N/A
Qu et al. (41)	RR	Experienced practitioners (*n* = 10)	Within-subject controls (relaxation)	Sudarshan Kriya and yoga	2 days for 2 h, 1.5 months to 5 years of experience	Genome-wide: Illumina Human WG-6 v3 Bead Chip, 9 genes: qPCR (Gene ontology)	Peripheral blood lymphocytes	All pathways ns	N/A
Dusek et al. (42)	CS + L	Experienced practitioners (*n* = 19)	Naïve (*n* = 20)	Relaxation response	20 min/day, 8 weeks	Genome-wide: Affymetrix HG U133 Plus (GSEA)	PBMC	CS: ubiquitin−, proteasome−, stress response−, ribosomal protein+ L: stress response−, metabolism−	N/A
Bhasin et al. (43)	CS + L + RR	Experienced practitioners (*n* = 26)	Naïve (*n* = 26)	Relaxation response	20 min/day, 8 weeks	Genome-wide:HR U133A (GSEA)	PBMC	ATP+, INS+, NF-kb− FeNO+	N/A
Kaliman et al. (44)	CS + RR	Experienced practitioners (*n* = 19)	Naïve (*n* = 21)	Mindfulness meditation	>30 min/day, 3 years	RT-PCR of 23 genes	PBMC	CS: ns RR: Inflammatory genes−, circadian genes ns, chromatin genes+, Cortisol−	Stress reactivity−
Epel et al. (45)	CS + L + RR	Experienced practitioners (*n* = 30)	Naïve (*n* = 33) Vacation (*n* = 31)	Meditation and yoga retreat	4 h of meditation and 3 h of yoga per day, 4 days	Genome-wide: RNA-Seq (Gene ontology)	PBMC	All groups: stress response−, wound healing−, injury− Experienced: protein synthesis−, viral expression−, viral infectious cycle− TNF− Aβ42/Aβ40+ Telomerase (experienced+, novice ns)	Depression−, stress−, vitality−, mindfulness−
Summary	CS = 17% LG = 50% RR = 11% Mixed = 22%	M (participants per group) = 23.55 Normal population = 50% Stressed populations = 50% (33% breast cancer + 22% caregivers + 45% others)	No control group = 22% Passive control = 11% Active control = 67% (50% naïve + 17% CBT + 17% HE + 6% others)	Mindfulness = 22% Relaxation response = 17% Other MBI = 61% Interventions with a physical component = 44%	46% of interventions lasted 8–12 weeks; 33% of interventions had only weekly meetings	44% of studies used TELiS and all found a downregulation of NF-κB	72% of studies did gene expression analysis from PBMC, 17% from lymphocytes	81% of studies found a reduction inflammation related genes and/or transcription factors	56% of studies did not measure any psychological outcomes

*Study design types used: Aβ, amyloid beta; CS, cross-sectional; L, longitudinal; RR, rapid response. Technologies and analysis used: RT PCR, real-time polymerase chain reaction; RNA-Seq, RNA sequencing; TELiS; Transcription Element Listening System; CBT, cognitive behavioral therapy; GSEA, Gene Set Enrichment Analysis. Cell types: PBMC, peripheral blood mononuclear cells. Transcription factors: GR, glucocorticoid receptor; IRF, interferon regulatory factor; NF-κB, nuclear factor kappa B; CREB, cAMP response element-binding protein; AP, activator protein; INS, insulin. Proteins: CRP, C-reactive protein; IL, interleukin; TNF, tumor necrosis factor; TLR, toll-like receptor. Other biological measures: FeNO, fractional exhaled nitric oxide; ESR, erythrocyte sedimentation rate*.

## Summary of Studies on MBIs Using Gene Expression Analysis

In Table 1, we summarize the biological and psychological outcomes for each study, including which genes were upregulated or downregulated, as well as the type of MBI, control group, and gene expression technology used. Below, we briefly describe the rationale and results for each study; for further details, please see Table 1.

### Cross-sectional Studies

#### Genomic Profiling of Neutrophil Transcripts in Asian Qigong Practitioners: A Pilot Study in Gene Regulation by MBI

The very first study of mind–body therapies that included gene expression compared gene profiles of six long-term Falun Gong Qigong practitioners and six healthy controls (29). Falun Gong is a form of Qigong that requires an intensive daily practice of 1–2 h, which includes reading spiritual Qigong books and a light physical activity in the form of meditative movement. Qigong practitioners had been using this technique from 1 to 5 years, every day from 1 to 2 h, while controls had been physically inactive for at least 1 year and did not practice any mind–body technique. In this study, gene expression analysis was carried out from neutrophils, which are the most prevalent type of white blood cells and are crucial in fighting infection. Using a microarray of 12,000 genes, they found that Qigong practitioners had 132 downregulated and 118 upregulated genes in comparison with controls. Some of the differentially expressed genes have common functions, thus the results suggest that Qigong enhances immunity, downregulates cellular metabolism, and delays cell death.

#### Gene Expression Profiling in Practitioners of Sudarshan Kriya (SK)

Sharma and colleagues (30) explored the effects of SK by comparing 42 long-term practitioners with controls who do not practice any MBI. SK is commonly practiced for 1 h per day and it consists of several breathing techniques, some of which are paired with movement. Controls were not only matched for age and sex but also for socioeconomic status, body mass index (BMI), diet, smoking, and alcohol consumption. Their hypothesis was that regular SK practice improves stress regulation, which should be reflected in gene expression. To test this, white blood cells were obtained from participants, but only a relatively small number of genes were analyzed. The nine genes of interest were related to oxidative stress, DNA damage, cell cycle control, aging, and cell death. Although psychological stress was not measured, based on the gene expression results researchers suggested that SK attenuates the effects of stress on cells due to an increase in expression of one gene, although four stress-related genes remained unchanged. Additionally, they concluded that SK enhances the immune system because it upregulates genes that inhibit cell death.

#### To Study the Effect of the Sequence of Seven Pranayama by Swami Ramdev on Gene Expression in Leukemia Patients and Rapid Interpretation of Gene Expression

Kumar and Balkrishna (31) conducted a study on the effects of seven breathing patterns developed by a popular Indian yoga teacher, Swami Ramdev. The sample consisted of eight patients with chronic lymphocytic leukemia, some of whom practiced breathing techniques, while others served as control. The exact number of participants per group is not reported, neither is the practice frequency. Surprisingly, the results showed that 4,428 genes (out of 28,000 analyzed) were upregulated up to twofold in leukemia patients who practiced breathing techniques. However, the published report lacks further details about used methods and procedures and it was not externally peer reviewed. Of 4,428 differentially expressed genes, only a set of upregulated stress-related genes is reported, along with upregulated pair of genes that delays cell death, which suggests improved immune regulation.

### Longitudinal Designs

#### Mindfulness-Based Stress Reduction (MBSR) Training Reduces Loneliness and Pro-inflammatory Gene Expression in Older Adults: A Small Randomized Controlled Trial

Creswell and colleagues (32) attempted to reduce loneliness in older adults as this is one of the leading risk factors for morbidity and mortality. Forty healthy older adults (age 55–85) were randomly assigned either to an 8-week MBSR course or to a wait-list control group. The MBSR course is a standardized program that consists of eight weekly 2-h meetings and one day-long retreat. Additionally, participants are expected to practice mindfulness every day at home for 30 min during the 8-week period. MBSR cannot be delivered effectively in large groups, so they formed three groups, each with a different teacher.

They tested if increased inflammation is a mechanism by which loneliness promotes disease in older adults. Inflammation was measured through changes in transcriptome and in protein markers of inflammation [C-reactive protein (CRP) and IL-6]. Blood samples were taken at baseline and after completion of MBSR. There also were self-report measures of loneliness and mindfulness. MBSR class attendances and minutes of daily home practice were measured as control variables.

Genome-wide transcriptional profiling was done from peripheral blood mononuclear cells (PBMCs) controlling for sex, age, ethnicity, BMI, as well as the contribution of sleep quality and exercise. Effect size cutoff of 25% was used in statistical analysis, which was followed with bioinformatics analysis of differentially expressed genes to see how many of them are targeted with NF-κB transcription factors (TELiS transcription factor search). They were interested in NF-κB because previous studies found that genes targeted with this transcription factor are more expressed in people who are lonely (46), which promotes inflammation.

At baseline, older adults who reported more loneliness showed higher expression of pro-inflammatory genes targeted with NF-κB transcription factor. After MBSR participants reported reduced loneliness and gene analysis showed a reversal of pro-inflammatory gene expression pattern. Further analysis showed that genes that changed expression originated mostly from monocytes and B lymphocytes. Regarding protein biomarkers of inflammation, there were no significant changes in CRP and IL-6 after MBSR.

#### Yogic Meditation Reverses NF-κB and IRF-Related Transcriptome Dynamics in Leukocytes of Family Dementia Caregivers in a Randomized Controlled Trial

Black and colleagues (33) did a study on a sample of people who were caring for a frail or demented family member. Caregivers tend to have worse mental and physical health than matched controls, probably due to stress-induced upregulation of inflammation-related genes and downregulation of innate antiviral genes. Previous studies found that interventions aimed at stress reduction improve immune functioning among caregivers, so Black and colleagues (33) wanted to explore molecular mechanisms by which inflammation is reduced. Twenty-three caregivers did a 12-min Kirtan Kriya Meditation (KKM) practice guided by an audio recording every day for 8 weeks. The practice starts with 1 min of mind and body awareness followed by chanting “birth, life, death, rebirth” in Sanskrit with accompanying hand gestures, and it ends with breathing deeply and visualizing light. The effects of KKM were actively controlled: 20 caregivers were listening to relaxing music with eyes closed for 12 min, every day for 8 weeks. The levels of depression and mental health were measured with questionnaires before and after the intervention and blood samples were taken to obtain PBMCs for transcriptional profiling. Gender, illness burden, and BMI were controlled for.

There was a significantly greater reduction of depressive symptoms and an improvement in mental health in the meditation group. Furthermore, 49 genes were downregulated and 19 upregulated in the KKM group in relation to the relaxation group. These differentially expressed genes were further analyzed with TELiS, which confirmed the hypothesis that there is a decrease in pro-inflammatory gene expression (related to NF-κB) and an increase in antiviral gene expression (IRF-1). This suggests that KKM improved the immune system in terms of inflammation reduction and creating better defense against viruses. Transcript origin analysis (TOA) found that most of the observed gene expression changes stem from B lymphocytes and plasmacytoid dendritic cells.

#### Tai Chi, Cellular Inflammation, and Transcriptome Dynamics in Breast Cancer Survivors with Insomnia: A Randomized Controlled Trial

Irwin and colleagues (34) explored the effects of Tai Chi, a Chinese practice that combines moderate exercise, deep breathing, and meditation, on inflammation and sleep in breast cancer survivors in comparison with cognitive behavioral therapy for insomnia [cognitive behavioral therapy (CBT)-I]. Both breast cancer and sleep deprivation are associated with inflammation, thus this sample had high levels of inflammation at baseline. The study adopted a multi-level approach to measuring effects of Tai Chi on inflammation that included systemic (circulating levels of CRP), cellular [toll-like receptor (TLR)-4-activated production of pro-inflammatory cytokines IL-6 and TNF], and genome-wide gene expression followed by bioinformatics analyses. Both Tai Chi and CBT groups had 40 participants who attended 2-h meetings once a week for 3 months. BMI and physical activity changes during interventions were controlled for, as they usually are associated with inflammation.

While CRP did not change after either of the interventions, IL-6 was marginally reduced and TNF was significantly reduced after Tai Chi, indicating that it can reduce cellular inflammatory responses. Similarly, gene expression analysis found a 9% reduction in expression of 19 pro-inflammatory genes and a 3.3% increase in expression of 34 genes involved in the production of proteins that regulate anti-viral response and tumor activity in the Tai Chi group relative to CBT-I. In total, 68 genes were downregulated and 19 upregulated after Tai Chi. The downregulated genes are involved in the generation of white blood cells and inflammation. Similar to previous studies, TELiS bioinformatics analysis found a significant reduction of activity of pro-inflammatory transcription factor NF-κB.

#### Yoga Reduces Inflammatory Signaling in Fatigued Breast Cancer Survivors: A Randomized Controlled Trial

Bower et al. (35) explored the effects of 3 months of Iyengar yoga on inflammatory processes in breast cancer survivors with fatigue. There were 16 people in the yoga group and 15 in the health education control group. Inflammation is associated with cancer and previous studies have found that breast cancer survivors with fatigue have higher levels of inflammation than non-fatigued breast cancer survivors (47). The hypothesis was that Iyengar yoga (a form of Hatha yoga with emphasis on precise alignment and breath control in each posture) would reduce inflammation-related gene expression, as well as decrease levels of circulating markers of inflammation.

Instead of measuring cytokines directly, Bower and colleagues chose downstream markers of pro-inflammatory cytokine activity, which are easier to detect as they are produced in a greater amount. The downstream markers are also considered a more accurate and stable measure of inflammation than the cytokines that produce them. Downstream markers included were as follows: sTNF-RII (a marker of TNF activity), IL-1ra, and CRP (markers of IL activity). These markers were measured from blood, while cortisol was measured from saliva (samples collected by participants themselves) immediately after waking, 30 min and 8 h after waking, and before bedtime.

Genome-wide transcriptional profiling identified 282 genes that were upregulated and 153 downregulated genes after 3 months of yoga. A 15% gene expression change was considered statistically significant, unlike other studies that set 20% as a cutoff point. The majority of downregulated genes were related to type I interferon responses (i.e., cytokines that are released when a virus infects a cell), which has previously been associated with fatigue in cancer patients. Similarly, behavioral measures of fatigue were significantly reduced after months of yoga and remained reduced at a 3-month follow-up.

Transcription Element Listening System showed that yoga reduced the activity of NF-κB, which is suggestive of lower inflammation, as this is a key regulator of pro-inflammatory gene expression. CREB is another transcription factor whose activity was reduced with yoga, suggesting a downregulation of the SNS. Finally, TELiS found that yoga increased the activity of anti-inflammatory glucocorticoid receptor (GR), which indicates a change in HPA axis in terms of responding better to cortisol and stopping the stress response more quickly. However, such change in the HPA axis should lead to reduced levels of cortisol, which was not verified with the cortisol analysis from saliva. Regarding downstream markers of inflammation, sTNF-RII increased in the control group, but remained at the same level in the yoga group. There were no significant changes for IL-1ra and CRP.

#### Mindfulness Meditation for Younger Breast Cancer Survivors: A Randomized Controlled Trial

Bower and colleagues (36) conducted another study, this time to assess cost-effectiveness of mindfulness intervention for women who had been diagnosed with early-stage breast cancer (from stage 0 to stage 3) before the age of 50 and had finished treatment from 3 months to 10 years ago. Mindful awareness practices (MAP) is a program similar to MBSR, but tailored for cancer survivors. It consists of six weekly 2-h group meetings and daily practice increasing from 5 to 20 min. Thirty-nine participants were assigned to MAP and 32 were assigned to a wait list. Unlike previous studies, Bower and colleagues used several psychological measures (stress and depression as primary outcomes; positive affect, intrusive thoughts, fear of recurrence, peace and meaning, and sleep quality as secondary outcomes) and measures of physical symptoms (fatigue, pain, and hot flashes). They examined gene expression changes in the whole genome and measured proteins related to increased inflammation (IL-6, CRP) and one protein related to cancer—soluble tumor necrosis factor receptor type 2 (sTNF-RII). Marital status, radiation treatment, smoking, and depressive symptoms differed between groups at baseline and were included as covariates in the data analysis. Additional covariates were minutes of meditation practice, time since diagnosis, chemotherapy, and endocrine therapy.

Mindful awareness practices significantly reduced stress, fatigue, sleep disturbance, hot flashes, and marginally reduced depressive symptoms. Conversely, it significantly increased positive affect and peace and meaning. A set of 19 pro-inflammatory genes was significantly downregulated with MAP when compared to the control condition. TELiS analysis of significantly changed genes found that transcription factor NF-κB showed a significant decrease while anti-inflammatory GR and interferon regulatory factors (IRF) increased and CREB remained the same. IL-6 was not significantly changed in general, but those who practiced meditation more frequently had lower levels of IL-6, while other proteins were non-significant even after the adjustment for the practice frequency. Downregulated genes mostly originated from monocytes and dendritic cells, while upregulated genes mostly originated from B lymphocytes.

#### Effects of Lifestyle Modification on Telomerase Gene Expression in Hypertensive Patients: A Pilot Trial of Stress Reduction and Health Education Programs in African Americans

Duraimani and colleagues (37) compared the effectiveness of a program that consisted of transcendental meditation and health education with a program of extensive health education alone in hypertensive adults. Extensive health education used lectures, videos, field trips, and social support to motivate participants to lose weight, adopt a healthy diet, exercise, eat less sodium, and drink less alcohol. Forty-eight hypertensive adults were randomly assigned to each group and attended weekly sessions for 4 months. Researchers only assessed the expression of two genes related to telomeres (hTR and hTERT), which are nucleotides at the end of chromosomes that shorten every time a cell divides and are associated with aging. They also measured blood pressure, lifestyle (diet and physical activity), and anger.

Both interventions equally increased the expression of telomerase-related genes. Telomeres themselves did not change with either intervention, though this rarely happens over the course of just a few months. Extensive health education proved to be better for hypertensive adults because it lowered diastolic blood pressure more than TM and led to healthier lifestyle behaviors.

#### CBT and Tai Chi Reverse Cellular and Genomic Markers of Inflammation in Late Life Insomnia: A Randomized Controlled Trial

Irwin and colleagues (34) conducted another study on the effects of Tai Chi, but this time on a sample of 120 older adults with insomnia. CBT-I was another experimental condition that was compared to Tai Chi, while a sleep seminar education was the control group. Each group consisted of 2-h weekly meetings over 4 months. As in the study described above (34), they adopted a multi-level approach to inflammation and measured CRP, TLR-4 activation of TNF and IL-6, and gene expression, while controlling for BMI and physical activity changes during interventions.

Behavioral outcomes regarding sleep are not reported in this paper. Four months after the intervention had finished, CRP was significantly reduced in the CBT-I group and remained at the same level after 16 months. In the Tai Chi group, CRP was only marginally reduced after 4 months and it became non-significant afterward. On the other hand, pro-inflammatory cytokines were reduced in both groups 2 months after the intervention, but they remained reduced for 16 months in the Tai Chi group alone. Gene expression profiling was carried out on a random subsample of 78 older adults at a 4-month follow-up. Relative to sleep education, CBT-I downregulated 347 genes and upregulated 191 genes, while Tai Chi downregulated 202 genes and upregulated 52 genes. The majority of downregulated genes after CBT-I and Tai Chi are involved in inflammation. On the other hand, the majority of upregulated genes after CBT-I are involved in interferon and antibody responses, while those upregulated after Tai Chi do not have a known common function. TELiS found that both CBT-I and Tai Chi reduced activity of NF-κB relative to sleep education, though the difference was only marginally significant in the Tai Chi group. Both interventions reduced activity of CREB as well, while Tai Chi also reduced activity of activator protein 1 (AP-1, controls cellular differentiation, proliferation, and cell death) and marginally increased GR activity. TOA found that the genes that are downregulated by CBT-I and Tai Chi originated mostly from monocytes and dendritic cells.

#### Genomic and Clinical Effects Associated with a RR MBI in Patients with Irritable Bowel Syndrome (IBS) and Inflammatory Bowel Disease (IBD)

Kuo et al. (38) undertook an uncontrolled trial with a mixed sample of 19 patients with IBS and 29 patients with IBD. Both IBS and IBD are chronic diseases of the digestive system that are exacerbated with stress, though they have different underlying physiology and symptoms. Previous studies found that psychological interventions such as psychotherapy and stress management can reduce symptoms and improve quality of life in IBD (48) and even more so in IBS (49). In this study, researchers explored if a relaxation response-based mind–body intervention (RR-MBI) could affect quality of life, inflammatory markers, and gene expression in IBS and IBD patients. The RR-MBI consisted of nine weekly meetings of 1.5 h and daily home practice of 15–20 min. The meetings included a variety of practical skills that induce the RR (e.g., breath focus, imagery, mindful awareness, and yoga) and cognitive skills that help to cope with stress. The theoretical part included lectures about the physiology of stress and digestion and promotion of health behaviors. Participants completed a set of self-report measures of common symptoms to both IBS and IBD (pain symptoms and catastrophizing, state and trait anxiety) and a set of disease-specific self-report measures (quality of life, severity of symptoms). Inflammation was measured as rate of sedimentation of red blood cells (erythrocyte sedimentation rate, ESR) and levels of CRP.

Immediately after RR-MBI and at a short-term follow-up 3 weeks later, both IBS and IBD patients showed greater quality of life and a significant reduction of symptoms of their condition and of anxiety. They reported improved coping with pain, but no change in how pain interferes with their functioning. Regarding biological measures, there was no change in ESR and CRP. In the IBD group, a total of 1059 genes had changed. These were related to improvements in inflammatory response, cell growth, proliferation, and oxidative stress-related pathways—kinases, inflammation, cell cycle, and proliferation. In the IBD group, 119 genes that are related to cell cycle regulation and DNA damage changed expression. Bioinformatics analysis of genes that changed expression (by using Interactive network analysis) found that NF-κB is a key molecule for both IBS and IBD.

#### Biomarkers of Resilience in Stress Reduction for Caregivers of Alzheimer’s Patients

Ho and colleagues (39) chose a sample of caregivers, a chronically stressed population, to test the effects of MBSR. The intervention was slightly modified by shortening the length of weekly classes from regular 2.5 to 1.5 h to meet the demanding daily schedules of caregivers, but the content remained the same. There was no control group in this study and the sample consisted of only 25 participants. Psychological outcomes were measured with a detailed Caregiver Self-Assessment Questionnaire (CSAQ) that consists of items about depression, burden, stress, grief and represents overall psychological distress. After MBSR, caregivers showed improvements on CSAQ that positively correlated with mindfulness score, which means that benefits were more pronounced in those that increased their levels mindfulness.

Based on the variability in the CSAQ score, researchers classified all 25 participants into three MBSR responder categories: poor responder, moderate responder, and good responder. This categorization was the basis for the gene expression analysis. Researchers identified 194 differentially expressed genes that can be used to predict to which responder category each caregiver belongs. These genes were related to inflammation, stress response, and depression, which suggest that psychological benefits of MBSR might be emerging due to reduction in these variables. Furthermore, researchers identified 91 genes that can be measured at baseline to predict with 94.7% accuracy the likelihood that a caregiver will get psychological benefits from MBSR. These genes were related to immune system functions, such as toll signaling and insulin, which suggests that the likelihood to benefit from MBSR depends on immunological status.

### Rapid Response

#### Genome-Wide Expression Changes in a Higher State of Consciousness

Ravnik-Glavač and colleagues (40) explored gene expression changes in two highly experienced practitioners (one with 23 years of experience and the other with 25) who claimed to occasionally move into a higher state of consciousness (a state of “pure awareness” without thoughts, feelings, or perceptions) that can last for several days after a single meditation session. They both practiced similar forms of meditation that stems from Buddhist traditions and aim to extend awareness (Zen, Kriya yoga, Kundalini yoga, and pranayama). Researchers obtained blood samples while meditators were in their “normal” state of consciousness, which was used as a control sample. When each of the meditators felt he entered a “higher” state of consciousness, he was invited to the lab to record electroencephalography (EEG) while he meditated. For this purpose, one participant practiced Zen and Kundalini meditation and the other meditated on mental quietness and a Buddha visualization. Blood samples were collected after meditation at the same time of the day (not more than 1.5 h apart) in order to control for circadian gene expression changes.

Electroencephalography showed almost identical patterns in both meditators: increased theta and alpha frequency range. Genes that changed expression for 30% or more after entering into higher consciousness were considered significant. For one participant, 1,688 genes changed expression (1,559 downregulated and 109 upregulated) and 608 for the other (338 upregulated and 270 downregulated). Although the number of changed genes differed between meditators, they shared 118 genes. The genes that changed in both meditators suggest a downregulation of metabolism and cell cycle processes. Additionally, some of the genes involved in immune system, cell death, and the stress response were downregulated. However, the two gene expression profiles were too different and thus difficult to compare and make generalizable conclusions.

#### Rapid Gene Expression Changes in Peripheral Blood Lymphocytes upon Practice of a Comprehensive Yoga Program

Qu et al. (41) were interested in rapid changes in gene expression that take place immediately after contemplative practice. Intervention consisted of gentle yoga postures, breathing exercises, and meditation, which they termed Sudarshan Kriya and related practices (SK&P). They had 10 participants, all of whom were recruited at a yoga retreat and their experience in SK&P ranged from 1.5 months to 5 years. In the first 2 days, participants practiced SK&P led by experienced teachers for 2 h. In the remaining 2 days, they had a walk in nature (to control for the physical aspect of yoga in SK&P) followed by listening to relaxing music (to control for the relaxation aspect of meditation and breathing exercise in SK&P), which lasted 2 h as well and was at the same time of the day. They were only interested in gene expression and no other measures were taken besides daily blood samples to obtain PBMCs. Gene profiles were compared for each participant before and after each day of practice. Hierarchical clustering showed that SK&P changed expression of threefold more genes than the control program: 111 genes after SK&P (54 upregulated and 57 downregulated), 38 after the walk and relaxing music (15 upregulated and 23 downregulated), and 14 genes were commonly affected by both interventions. Thirty-six percentage of the genes that were changed after walking and relaxing music were also changed after SK&P, which suggests that a yoga program has more benefits in addition to those provided by physical activity and relaxation. Although there were many significant gene expression changes, bioinformatics analysis (by using different methods of gene ontology analysis) did not find a significant pathway (e.g., NF-κB as commonly found in other studies).

### Mixed Designs

#### Genomic Counter-Stress Changes Induced by the RR

The RR has been defined as a physiological state that represents the opposite state of the stress response (50). It is characterized by decreased oxygen consumption and carbon dioxide elimination, reduced blood pressure, and heart and respiration rate. RR is elicited by focusing on a word, phrase, sound, or movement while attempting to disengage from thoughts. Meditation is just one of the many methods to induce the RR, along with yoga, Tai Chi, Qi Gong, breathing exercises, meditation, progressive muscle relaxation, and repetitive prayer. Beneficial clinical effects of the RR have been amply reported [for a review see Ref. (51)], and in this cross-sectional study, researchers explored differences in gene expression that occur with regular practice of this MBI (42). First, they compared long-term practitioners (*n* = 19) with age- and gender-matched controls and found differences in the expression of 2,209 genes (1,275 upregulated and 934 downregulated). Then the control group (*n* = 20) went through 8 weeks of RR training and the analysis of differences in their gene profiles before and after training identified that 1,561 of genes had changed expression (874 upregulated and 687 downregulated). However, there were significant overlaps of differentially expressed genes among all three groups: only 595 of 2,209 genes that were changed in long-term practitioners were unique to this group. Bioinformatics analyses showed that long-term practitioners presented a downregulation of ubiquitin, proteasome, and stress response, an upregulation of ribosomal protein, and mixed directions of change in apoptosis and immune system. On the other hand, 418 of 1,561 genes were changed with short-term practice only—when naïve participants went through 8 weeks of RR practice, there was a significant enrichment of gene sets related to stress responses and metabolism. This means that short-term and long-term RR practice may lead to distinct gene expression changes.

The results were validated in a separate independent analysis on a new set of samples derived from previous groups (five controls, five short-term practitioners, and six long-term practitioners). Validation results were similar to the original results from the full sample, which supports the assumption that these changes do not occur randomly.

#### RR Induces Temporal Transcriptome Changes in Energy Metabolism, Insulin Secretion, and Inflammatory Pathways

Bhasin and colleagues (43) explored differences in gene expression changes after one RR session in expert meditators and novices. They assessed both long- and short-term effects of the RR. Expert participants had between 4 and 20 years of experience in RR, while novices did not have any experience and undertook the RR training as a part of the study intervention; this consisted of eight weekly private sessions with an experienced clinician and a 20-min audio recording with an RR sequence for daily home practice. The RR sequence consisted of diaphragmatic breathing, body scan, mantra repetition, and mindfulness meditation. Both experts and novices listened to the same audio recording in a laboratory session. Prior to the RR training, novices listened to a health education audio of the same length that served as a control intervention. In both cases, blood samples were obtained at three time points: before, immediately after, and 15 min after listening to the audio recording. The only outcome measures were gene expression and the amount of fractional exhaled nitric oxide (FeNO), which influences blood pressure. Results showed that more genes were changed in experts than in short-term practitioners or novices and that the group difference was the most pronounced 15 min after the RR. They then proceeded to undertake various analyses, including Molecular Functions Enrichment Analysis and Gene Set Enrichment Analysis.

Results showed that experts and short-term practitioners had different gene expression profiles at baseline. Following a RR session, experts showed more consistent and pronounced gene expression changes than short-term practitioners. Both experts and short-term practitioners presented changes that have been linked to energy metabolism, electron transport chain, biological oxidation, and insulin secretion—all these pathways are crucial for mitochondrial energy mechanics, oxidative phosphorylation, and cell aging. Using systems biology analysis, it was found that the most upregulated critical molecules were ATP synthase and insulin, which promote mitochondrial energy production and utilization (resilience), and the most downregulated NF-κB pathway genes. Changes were generally more pronounced in experts. Upregulated genes were related to energy metabolism, mitochondrial function, insulin secretion, and telomere maintenance. Downregulated genes were related to inflammatory response and stress pathways. Finally, FeNO was increased or showed a trend toward increase during RR in all practitioners regardless of experience.

#### Rapid Changes in Histone Deacetylases and Inflammatory Gene Expression in Expert Meditators

Kaliman and colleagues (44) explored immediate effects of an intensive 8-h mindfulness meditation retreat in experienced meditators on the expression of three sets of genes with common functions (7 circadian, 10 chromatin modulatory, and 6 inflammatory genes) and on stress reactivity in a laboratory-induced stressful situation. Experienced meditators were compared to a control group with no meditation experience who had engaged in leisure activities of the same length. Against their hypotheses, they found no differences in the tested genes between expert and naïve groups before the meditation, but after the intervention there was a significant silencing of two out of six pro-inflammatory genes (RIPK2 and COX2) in experienced meditators only. Additionally, there were significant changes in the global modification of histones (H4ac and H3K4me3) and silencing of several histone deacetylase genes (HDAC 2, 3, and 9), all of which regulate the activity of other genes. The extent to which pro-inflammatory genes were silenced was associated with faster cortisol recovery to social stress. On the other hand, the expression of circadian genes was not affected with intensive mindfulness meditation.

#### Meditation and Vacation Effects Have an Impact on Disease-Associate Molecular Phenotypes

Epel and colleagues (45) were primarily interested in the effects of a 6-day residential retreat on people who did not have experience with meditation, but they wanted to control for what they called “the vacation effect.” They considered that when people go on retreats, they are not only meditating but are also away from the demands of their daily lives, which should significantly lower stress levels and change gene expression. They thus used an active control group that resided at the same location for the same amount of time, but without participating in any meditation or relaxing programs offered by the retreat center. The other group consisted of people who were new to meditation and who attended a 4-day intensive program of mantra meditation (4 h/day), yoga (3 h/day), lectures, and self-reflective exercises. Additionally, to be able to contrast the effects of a 4-day intensive meditation on novice meditators with experienced meditators, a group of regular meditators attended the same retreat. Psychological outcomes were depression, stress, vitality, and mindfulness—all of which improved for all groups after the intervention and remained positively changed at a 1-month follow-up. After a 10-month follow-up, novice meditators had less depressive symptoms than the vacation group, which suggests that learning meditation may have psychological benefits that last longer than those of merely going on a holiday.

The central biological outcome of this study was gene expression. There were 390 genes that changed expression in all three groups, most likely due to the relaxation component that was common to all groups. These gene expression changes referred primarily to lower expression of genes related to stress response, wound healing, and injury. In addition to these changes common across groups, regular meditators showed lower expression of genes involved in protein synthesis, viral expression, and viral infectious cycle, while the novice meditators had no distinctive gene expression changes.

They also assessed other biological outcomes, including telomerase (an enzyme that can stabilize or lengthen telomeres), TNF alpha, and amyloid beta (Aβ) metabolism. Greater ratio of proteins Aβ42/Aβ40 is associated with lower risk of dementia (52) depression (53), and mortality (54). The vacation group had significantly more TNF- than regular meditators and marginally more than novice meditators, which suggests an acute inflammatory response, possibly due to sun exposure or exercise. All groups in this study had a higher Aβ42/Aβ40 ratio after the retreat, most likely due to relaxation. An unexpected finding was that regular meditators had shorter telomeres, which is associated with aging, diabetes, cardiovascular disease, and some types of cancer.

## Discussion

The 18 examined studies indicate that MBIs reverse skewing of the transcriptome that is related to adversity, which counteracts the effects of stress on the immune system. Although most genes showed small or moderate effect sizes individually, a general pattern emerges: pro-inflammatory genes and pathways get downregulated (see Table 1 for a summary). Most studies (81%) that measured the activity of inflammation-related genes and/or NF-κB, a key transcription factor that controls the expression of inflammation-related genes, found a significant downregulation. The exceptions were two uncontrolled trials that measured the immediate effects of MBIs in experienced practitioners, which is probably the consequence of the sample sizes of 10 and 2 (40, 41), the norm being that 15 participants per group are necessary to provide statistical power greater than 80% for the gene expression outcome (32, 33). A further exception was one controlled trial that compared 19 long-term practitioners to 20 short-term practitioners of RR and did not detect changes in inflammatory pathways (42). This could be due to the different methods of gene expression detection and analysis, as all studies that employed genome-wide profiling followed by TELiS bioinformatics analysis consistently found a downregulation of NF-κB. Therefore, the results of the reviewed studies tentatively suggest that the various psychological and physiological benefits of MBIs may be mediated through the downregulation of pro-inflammatory genes and pathways. However, for this research to be able to show with greater confidence that the level of effectiveness of MBIs is predicated on these genetic expression changes, we need to address the severe limitations of the reviewed studies.

A major shortcoming of the literature is the lack of active control groups that carefully mirror the MBIs (e.g., length of time, meaningfulness of the practice). This should be a mandatory procedure in studies of gene expression analysis with behavioral interventions to account for the many non-specific effects of MBIs, such as social support or teacher–student relationship. An active control group was included in six out of the nine randomized controlled studies, but control conditions ranged from relaxation—which produces similar effects to MBIs regarding stress reduction—to education. Black and colleagues (33) probably achieved the most balanced solution, as both the meditation and the relaxation control group practiced at home with an audio CD of the same length of time and with eyes closed, thus making both conditions very similar. The effects of MBIs depend to a great extent on the amount of regular practice, but most studies did not measure practice frequency, simply assuming high adherence. Only two studies (32, 36) controlled for the frequency of practice in their gene expression analysis and found that some biological results became significant, when those individuals that practiced regularly were analyzed separately. It is important that future studies measure practice frequency and report dosage-dependent effects in addition to overall effects of MBIs on gene expression.

One other problem to consider are the various environmental and lifestyle factors that may change gene expression in similar ways to MBIs. For example, similar differences can be observed when analyzing gene expression from peripheral blood mononuclear cells (PBMCs) after exercise. Although at first there is an increase in the expression of pro-inflammatory genes due to regeneration of muscles after exercise, the long-term effects show a decrease in the expression of pro-inflammatory genes (55). In fact, 44% of interventions in this systematic review included a physical component, thus making it very difficult, if not impossible, to discern between the effects of MBIs from the effects of exercise. Similarly, food can contribute to inflammation. Diets rich in saturated fats are associated with pro-inflammatory gene expression profile, which is commonly observed in obese people (56). On the other hand, consuming some foods might reduce inflammatory gene expression, e.g., drinking 1 l of blueberry and grape juice daily for 4 weeks changes the expression of the genes related to apoptosis, immune response, cell adhesion, and lipid metabolism (57). Similarly, a diet rich in vegetables, fruits, fish, and unsaturated fats is associated with anti-inflammatory gene profile, while the opposite has been found for Western diet consisting of saturated fats, sugars, and refined food products (58). Similar changes have been observed in older adults after just one Mediterranean diet meal (59) or in healthy adults after consuming 250 ml of red wine (60) or 50 ml of olive oil (61). However, in spite of this literature, only two of the studies we reviewed tested if the MBIs had any influence on lifestyle (e.g., sleep, diet, and exercise) that may have explained gene expression changes.

Another limitation is inherent to gene expression data. By themselves these do not provide much useful information unless the relationship between gene expression and psychological variables is directly explored. Only two of the reviewed studies (32, 44) attempted to find associations between gene expression changes and psychological constructs, such as stress reactivity and loneliness. Four other studies (33, 36–38) included psychological measures, but only to test the efficacy of their interventional programs, not to interpret observed gene expression differences. The majority of studies (56%) only included biological outcomes, which reveal a dire need for interdisciplinary collaborations in order to fully understand the interaction between molecular and psychological changes associated with MBIs.

The studies presented considerable variation, both in their type of interventions and gene expression assessment. MBIs varied from seated meditation at home to movement in groups, with lengths ranging from 4 days to 4 months: half of them used healthy adults while the other half had clinical or highly stressed samples. One interesting hypothesis to test is that the effects of MBIs will be easier to detect on populations with high levels of inflammatory gene expression at baseline [such as older adults; (32)], though this remains to be tried out in future studies, as there are no present data that allow us to compare the effect sizes of gene expression changes in different population.

Another source of heterogeneity in the reviewed studies is the cell type from which gene expression data are collected. In 72% of reviewed studies, data were obtained from peripheral blood mononuclear cells (PBMCs). As PBMCs consist of particular cell subtypes that have different gene expression patterns and functions, their variety could affect data interpretation. The results of studies that analyzed from which cell types the observed gene expression emerged (TOA) were mixed, thus all PBMCs will have to be included in future studies.

Another aspect to bear in mind is that the biological consequences of the observed gene expression changes were not found directly, because the studies that employed circulating proteins (e.g., CRP, interleukins, or cortisol) generally did not find significant results. In fact, 38% of the reviewed studies measured at least one inflammatory protein and the results were non-significant in 76% of cases and those that were significantly changed (usually TNF, CRP, and IL-6) are not consistently reduced across studies. Our systematic review indicates that circulating proteins rarely change after a few months of practice, which is how long the studies usually last (46% of studies in this review lasted between 6 and 12 weeks). This suggests that as long as the study interventions consist of only a few months of practice, it will be of limited value to measure proteins. Fortunately, gene expression is more sensitive to MBIs than circulating proteins. Gene expression changes are observed after a few weeks of meditation [e.g., Ref. (32, 33)], but they possibly emerge even after just a few days of intervention. Therefore, the conclusion that MBI techniques improve immune system function is made indirectly using bioinformatics analyses, which are based on previous studies from other areas that found associations between genes and immune outcomes (62).

One final methodological concern has to do with the assessment of inflammation. Throughout this review we encountered 11 different measures of inflammation. Thus, if a single inflammatory measure has decreased after an intervention, we cannot confidently conclude that the immune system is enhanced. Future studies should attempt to directly find functional consequences of observed gene expression changes. For instance, PMBC subtypes could be isolated before and after MBIs to verify if they show different *in vitro* responses.

Before widely integrating MBIs in health care, more research must be done with the aim of constructing and validating a comprehensive theory of MBIs with a multi-level approach that draws connections between genetic and other data, particularly psychological and behavioral. This is the only way of advancing the literature on MBIs and responding to recent criticisms about the theoretical incongruence and lack of consistent evidence for the benefits of these techniques [e.g., Ref. (63)]. Although the studies reviewed here provide preliminary evidence that MBIs are associated with a reduced risk of inflammation-related diseases, it is unclear whether they are more effective than a range of lifestyle changes commonly recommended as a part of healthy lifestyle, such as regular exercise and a Mediterranean diet.

## Conclusion

The results of 18 studies that used gene expression analysis in research on meditation and related MBIs have overall found downregulation of NF-κB-targeted genes, which can be understood as the reversal of the molecular signature of the effects of chronic stress. Even though the study designs, the population, and the types of MBI used in the studies included in this review vary, it indicates that some of the psychological and physical benefits of MBIs are underpinned by biological changes in NF-κB genes. These results need to be replicated in larger samples and with stronger research designs that control for non-specific effects of these practices and for as confounding lifestyle factors, such as sleep, diet, and exercise. This research opens the doors to the development and testing of a multi-level theory of MBIs, which integrates the biological, psychological, and environmental levels.

## Author Contributions

IBu: database search, study design, data interpretation, drafting the paper, final approval, agreement to be accountable. MF and IBr: study design, data interpretation, critical revision, final approval, agreement to be accountable. JJ and CM: data interpretation, critical revision, final approval, agreement to be accountable.

## Conflict of Interest Statement

The authors declare that the research was conducted in the absence of any commercial or financial relationships that could be construed as a potential conflict of interest.

## Appendix

**Table A1:** Common abbreviations and their definitions.

BMI	Body mass index, calculated as the body weight divided by square of the body height
CBT	Cognitive behavioral therapy, the most common approach in psychotherapy
CREB	cAMP response element-binding protein, a transcription factor that regulates genes involved in neuroplasticity and memory
CRP	C reactive protein, a protein produced by the liver that can be measured from the blood as an indicator of inflammation
CTRA	Conserved transcriptional response to adversity, a molecular signature of stress
FeNO	Fractional exhaled nitric oxide, a measure of airway inflammation
GR	Glucocorticoid receptor, a transcription factor that affects inflammation and cellular proliferation
GSEA	Gene Set Enrichment Analysis, a type of bioinformatics analysis of the genes that change expression
HPA	Hypothalamus–pituitary–adrenal (axis), the key circuitry involved in the stress response
IBD	Inflammatory bowel disease, a term that include Crohn’s disease and ulcerative colitis, which are chronic inflammatory disease of the colon and/or small intestine
IBS	Irritable bowel syndrome, a chronic inflammatory condition that brings symptoms such as cramps, bloating, diarrhea, or constipation
IL	Interleukin, a protein that regulates immune responses
IPA	Ingenuity Pathway Analysis, a type of bioinformatics analysis of the genes that change expression
IRF	Interferon regulatory factors, a family of transcription factors that regulate antiviral responses
KKM	Kirtan Kirya Meditation, a form of meditation that includes singing a mantra and finger gestures (mudras)
MAP	Mindful awareness practices, a standardized 6-week mindfulness intervention
MBCT	Mindfulness based cognitive therapy, an approach that combines CBT and mindfulness
MBI	Mind-body intervention, an umbrella term for meditation, yoga, mindfulness, Tai Chi, Qigong and relaxation response
MBSR	Mindfulness Based Stress Reduction, a standardized 8-week mindfulness intervention
miRNA	microRNA, a small non-coding RNA molecule that can interfere with the expression of a gene after it is transcribed
mRNA	Messenger RNA, a large family of RNAs that transport genetic infomation from DNA to ribosomes
NF-κB	Nuclear Factor Kappa B, a transcription factor regulating a large number of genes related to immune response, cell survival, differentiation, and proliferation
PBMC	Peripheral blood mononuclear cells, blood cells that have a round nucleus: lymphocytes and monocytes
RR	Relaxation response, it refers to any practice that can elicit a physiological response that is the opposite of the stress response
RT qPCR	Real time quantitative polymerase chain reaction is a laboratory technique that detects gene expression
SK&P	Sudarshan Kirya and related practices include yoga poses, breathing exercises and meditation
SNS	Sympathetic nervous system, activates fight or flight response when stress is detected
TELiS	Transcription Element Listening System, a type of bioinformatics analysis of the genes that change expression
TLR	Toll-like receptor, protein that plays a role in the immune system
TM	Transcendental meditation, a form of meditation based on the repetition of a mantra
TNF	Tumor necrosis factor, a prototypical pro-inflammatory cytokine that plays a central role in inflammation, immune system development, and apoptosis
TOA	Transcript origin analysis, a bioinformatics analysis that determines the cellular origin of detected gene expression changes
TSST	Trier Social Stress Test, a behavioral test of stress reactivity that consists in giving a speech and doing arithmetic operations in front of judges
